# How territoriality and sociality influence the habitat selection and movements of a large carnivore

**DOI:** 10.1002/ece3.11217

**Published:** 2024-04-16

**Authors:** K. Whitney Hansen, Nathan Ranc, John Morgan, Neil R. Jordan, J. Weldon McNutt, Alan Wilson, Christopher C. Wilmers

**Affiliations:** ^1^ Environmental Studies Department University of California Santa Cruz California USA; ^2^ Botswana Predator Conservation Maun Botswana; ^3^ Université de Toulouse, INRAE, CEFS Castanet‐Tolosan France; ^4^ Center for Ecosystem Science University of New South Wales Sydney New South Wales Australia; ^5^ Taronga Conservation Society Australia Dubbo New South Wales Australia; ^6^ Structure & Motion Lab, Comparative Biomedical Sciences Royal Veterinary College London UK

**Keywords:** habitat selection, *Lycaon pictus*, movement ecology, sociality, territoriality

## Abstract

While territoriality is one of the key mechanisms influencing carnivore space use, most studies quantify resource selection and movement in the absence of conspecific influence or territorial structure. Our analysis incorporated social information in a resource selection framework to investigate mechanisms of territoriality and intra‐specific competition on the habitat selection of a large, social carnivore. We fit integrated step selection functions to 3‐h GPS data from 12 collared African wild dog packs in the Okavango Delta and estimated selection coefficients using a conditional Poisson likelihood with random effects. Packs selected for their neighbors' 30‐day boundary (defined as their 95% kernel density estimate) and for their *own* 90‐day core (defined as their 50% kernel density estimate). Neighbors' 30‐day boundary had a greater influence on resource selection than any habitat feature. Habitat selection differed when they were within versus beyond their neighbors' 30‐day boundary. Pack size, pack tenure, pup presence, and seasonality all mediated how packs responded to neighbors' space use, and seasonal dynamics altered the strength of residency. While newly‐formed packs and packs with pups avoided their neighbors' boundary, older packs and those without pups selected for it. Packs also selected for the boundary of larger neighboring packs more strongly than that of smaller ones. Social structure within packs has implications for how they interact with conspecifics, and therefore how they are distributed across the landscape. Future research should continue to investigate how territorial processes are mediated by social dynamics and, in turn, how territorial structure mediates resource selection and movement. These results could inform the development of a human–wildlife conflict (HWC) mitigation tool by co‐opting the mechanisms of conspecific interactions to manage space use of endangered carnivores.

## Graphical abstract

We incorporated intra‐specific competition in a resource selection framework, via territorial data of conspecifics, to investigate mechanisms of territoriality and to better understand the role of neighboring packs on African wild dog habitat selection. Social structure within packs has implications for how they interact with conspecifics, and therefore how they are distributed across the landscape. Future research should continue to investigate how territorial processes are mediated by social dynamics and, in turn, how territorial structure mediates resource selection and movement.

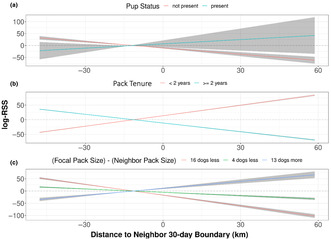

## 1 INTRODUCTION

The decisions animals make about where and how to move impact both their individual survival and species distributions (Nathan et al., 2008). Studying the mechanisms that govern animal space use improves our understanding of ecological processes and expands our capacity to design effective conservation strategies to ensure species persistence (Morales et al., 2010; Nathan et al., 2008). Territoriality is a widespread phenomenon in vertebrates and is one of the key mechanisms underlying animal movement (Maher & Lott, 2006). In carnivores, the proximate causes of territoriality vary by species, from exclusive resource ownership, sociality, population dynamics, reproductive strategy, or some combination of these factors (Maher & Lott, 2006). For example, the resource dispersion hypothesis (RDH) and intruder pressure hypothesis (IPH) are two leading hypotheses for explaining proximate causes of sociality in carnivores, and link territoriality and sociality to resource distribution (Eloy, 2003; Marneweck et al., 2019). RDH predicts that groups will maintain the smallest territory size needed to defend exclusive rights to a minimum number of patches that satisfies energetic costs of defense (Macdonald et al., 2015), while the IPH predicts larger territories should experience fewer intrusions from conspecifics and larger territory overlap in areas of low resource dispersion (Marneweck et al., 2019). Despite these theories linking resource distribution, territoriality, and social composition, little is known about how competitive interference influences the movement and resource selection of animals. By understanding the behavioral mechanisms underlying territorial movement we can potentially better manage large carnivores and their conflict with people (Wittemyer et al., 2019).

While most resource selection studies have not incorporated density dependent information in their models, ecological theory predicts that conspecifics should impact habitat selection through intra‐specific competition (Avgar et al., 2020). An established population of territorial animals such as carnivores would display an Ideal Despotic Distribution (IDD), whereby higher quality habitat is controlled by more dominant individuals and competition would strongly influence habitat selection (O'Neil et al., 2020). In an IDD, competitive interference reduces the quality of preferred habitat (Morris, 1994); the distribution of neighboring conspecifics in relation to available habitat, and their competitive abilities, will alter habitat preference and territorial structure of individuals (Sells & Mitchell, 2020). Despite these predictions, few studies on mammals have evaluated how conspecifics influence habitat selection (Buxton et al., 2020). By quantifying the space use of co‐occurring groups, we can incorporate a proxy for conspecific influence into our analysis and quantify the influence of neighboring conspecifics on the resource selection and movement behavior of territorial animals. Furthermore, by incorporating the social composition of conspecifics—e.g., group size and breeding status—in habitat selection, we can investigate which social parameter influences pack competitive ability. Social parameters boosting pack competitive ability are generally important aspects to survey for the long‐term survival of animals (Woodroffe et al., 2019).

Unraveling the mechanisms underlying space use and conspecific interaction is especially valuable to the development of biologically‐relevant human–wildlife conflict (HWC) mitigation tools, where territorial signals have the potential to be used to mimic residence and deter carnivores from livestock farming areas (Apps et al., 2013; Jackson et al., 2012).

Given the seasonal dynamics of their biology (Woodroffe et al., 2017), and the complex social dynamics both between and within packs, the African wild dog is perfectly suited to the study of how the interplay between sociality and territoriality influences animal movement and habitat selection. The African wild dog (*Lycaon pictus*) is a territorial carnivore forming large packs of up to 30 individuals (Creel et al., 2004). Packs are comprised of adult male and females with typically one reproductively active pair (the dominants), other related adults that assist in pup rearing and resource acquisition, and include the offspring of the dominants that may disperse after 2–3 years (Creel et al., 2004; McNutt, 1996). In the Okavango Delta, Botswana, packs average 10.4 individuals and maintain large territories (739 ± 81 km^2^; Pomilia et al., 2015) through scent marking, which includes the use of latrines (Claase et al., 2022), and infrequent inter‐pack fighting (Creel et al., 2004; Jordan et al., 2017). Their large territories and degree of territorial overlap are likely a result of multiple combining factors, such as the high lion density and low resource dispersion in the study area (Marneweck et al., 2019). Despite this overlap, packs strongly avoid direct confrontation with conspecifics from neighboring packs through their system of scent communication (Claase et al., 2022). Both interpack social attributes, such as kinship and relative pack size, and intrapack social attributes, such as pack tenure, pup presence, and breeding status, may mediate degree of territorial overlap (Jackson et al., 2017). During the annual denning season (June to September), wild dogs greatly restrict their movements (Pomilia et al., 2015; Woodroffe et al., 2017) but continue to engage in territorial monitoring and scent marking behaviors (Claase et al., 2022).

Existing mechanistic movement models have demonstrated how scent marking behavior can give rise to territoriality (Moorcroft et al., 2006), and how memory, kinship, and group size mediate territory formation (Bateman, 2014; Ellison et al., 2020; Moorcroft et al., 2006; Potts & Lewis, 2014). While fundamental to advancing our knowledge of territorial behavior, these studies have not evaluated how social interactions could alter habitat selection, nor its seasonal dependency. We also do not know how aspects of intra‐pack sociality, such as pack tenure or pup presence, might alter how packs respond to one another. In this study, we investigate the influence of territoriality and pack composition on habitat selection and movement of African wild dogs by integrating a proxy for intra‐specific competition in a resource selection framework. Here we fit a mechanistic movement model, the integrated step selection function (iSSF) (Avgar et al., 2016), to the GPS data and demographic field observations from 12 packs in the Okavango Delta to (1) evaluate the influence of territoriality on movement and selection, (2) quantify how habitat selection differs across territorial space, and (3) determine how seasonality and sociality of wild dogs—specifically pack size, pack tenure, and pup presence—mediate territorial processes.

## 2 MATERIALS AND METHODS

### 2.1 Study area

This study took place in the southwestern Okavango Delta of Botswana (study site is ca. 2600 km^2^; 19°31′ S, 23°37′ E; elevation ca. 950 m). The study area is composed of multiple habitat types, mainly floodplains, grasslands, savannah woodlands, and shrublands, some of which vary seasonally according to the Delta's flooding schedule (McNutt, 1996). The Delta's rainy season lasts from December to March, while the early flood season lasts from April to July and cumulates in a peak flooding season from August to November, due to the downflow of rains that fell higher up the catchment in the preceding wet season (Bennitt et al., 2014). In the Okavango Delta African wild dogs coexist with many other large carnivore species (lion [*Panthera leo*], leopard [*Panthera pardus*], spotted hyena [*Crocuta crocuta*], brown hyena [*Parahyaena brunnea*], and cheetah [*Acinonyx jubatus*]), and a wide variety of ungulates (Rich et al., 2016). See McNutt (1996) for further details.

### 2.2 Movement and demographic data

Botswana Predator Conservation (BPC) has been monitoring the subpopulation of African wild dogs in the study area since 1989. Between 2011 and 2022, 26 free‐ranging wild dogs from 19 different packs were fitted with GPS collars which were programmed to collect fixes either based on wild dog activity (Hubel et al., 2016; Wilson et al., 2013) or every 3 h. Collars were preferentially fitted to resident dominant/breeding individuals, allowing us to avoid potential dispersal forays and assume individual movement data reflects pack movement (Jordan et al., 2014). We used a combination of quality indicators measured by the activity‐based collars and a procedural investigation of the distances and time between GPS fixes (Urbano & Cagnacci, 2014 Chapter 8) to clean and sort GPS data into a single trajectory per pack. Given the uneven spread of collared individuals per pack, we transformed individual‐level trajectories into a single pack‐level trajectory. Data were regularized to a 3‐h resolution using the R package *amt* (Signer et al., 2019).

BPC's long‐term, exhaustive database of observed wild dogs in the study area classifies individuals by unique pelage patterns. Each sighting in the study area is geo‐tagged, timestamped, and associated with pack‐specific information including pack size, breeding status, and composition (e.g., a list of all individual wild dogs present). We matched the closest timestamped sighting (within 40 days) to the relevant GPS data. If no sightings occurred within 40 days of collected GPS data, these data were used without associated social information. We restricted pack trajectories to those which had a minimum of 100 complete steps, or 12 days, leaving us with 12 total packs (mean number of steps = 285, or approximately 36 days).

### 2.3 Modeling framework

By fitting both movement and resource selection processes to data, iSSF's allow for mechanistic inferences into how animals use space (Avgar et al., 2016). We fit iSSF's to estimate the coefficients which predict resource selection and movement metrics of wild dog packs, testing for territorial and social modifiers (Avgar et al., 2016). The iSSF allows for inference on movement behavior in addition to habitat selection by modifying the resource selection analysis (RSA) framework to simultaneously model step length and turning angle parameters (Avgar et al., 2016). To conduct our iSSF, we generated 20 control points per relocation by sampling step lengths and turning angles from distributions fit to our movement data (Avgar et al., 2016). Step lengths were drawn from a gamma distribution, and turning angles were drawn from a von Mises distribution. We estimated model coefficients using a conditional Poisson likelihood (Muff et al., 2020). Assuming there are i=1,…,I packs occurring at times $t=1,\ldots ,T_{i}$, each pack will have $j=1,\ldots ,J_{\mathrm{it}}$ locations per pack i per relocation t. Following Muff et al., (2020), we used a generalized linear mixed effects model using the R package *glmmTMB* (Magnusson et al., 2016). We modeled our data y, comprised of used and control locations, using a Poission distribution such that,

$$y_{\mathrm{ijt}}\sim \text{Poisson}\left(\lambda_{\mathrm{ijt}}\right)$$

with $\lambda_{\mathrm{ijt}}=\text{logit}\left(\alpha_{\mathrm{it}}+{\beta^{T}}_{\mathrm{ijt}}*x_{\mathrm{ijt}}\right)$where $\alpha_{\mathrm{it}}$ is a stratum‐specific intercept per pack i at time t (i.e., for each animal‐step), and cancels out when solving for the probability that $y_{\mathrm{ijt}}$ = 1, which is the probability a given point was used by a pack (Muff et al., 2020). $\beta^{T}$ is a vector of transposed covariates β, and $\;x_{\mathrm{ijt}}$ is a vector of covariate data for pack i at location j at time t. Selection covariates were included as random effects to account for inter‐pack variability in resource selection, differences in resource availability across territories, and to generate more accurate estimates and confidence intervals (Muff et al., 2020).

### 2.4 Habitat and movement covariates

To account for known habitat preferences while testing for territorial and social modifiers, we assigned each relocation and control point distance‐to‐land‐cover data values. Land cover data included roads, human settlements, bodies of water (seasonal pans and permanent water bodies), and four vegetation types—grassland, floodplain, mixed woodland, and mopane woodland (in equation 1 a vector of land cover data is denoted as $h\left(x_{\mathrm{tij}}\right)$; Bennitt et al., 2014). Covariates used to test selection were based on the habitat value at the end‐of‐step location. All covariates were standardized (mean‐centered and scaled by standard deviation), and tested for multicollinearity using Pearson's correlations. Only terms with a correlation value of |*r*| < .6 were included (Hinkle et al., 2003).

To jointly infer movement and habitat selection, step length ($l_{\mathrm{ijt}})$ and the natural log of step length ($\ln\left(l_{\mathrm{ijt}}\right)$) were included as movement terms in our habitat selection model (Avgar et al., 2016). We also included the cosine of the turning angle between each used/control point and the previous used point to model directional persistence ($\cos\left(\gamma_{\mathrm{ijt}-1}–\gamma_{\mathrm{ijt}}\right),$ where γ is the direction of movement between the current location and the previous one). We predicted that packs would have greater recursions in the denning as compared to non‐denning seasons, given that packs must make hunting forays and return to their dens multiple times per day; to this end, points were assigned a seasonal classification ($S_{\mathrm{ijt}}$; denning season June–September, pre‐denning season February–May, and post‐denning season October–January [Pomilia et al., 2015]) which we interacted with directional persistence. We also assigned each point a time of day classification ($D_{\mathrm{ijt}}$; daytime, nighttime, or crepuscular hours designated using R package *suncalc*) to account for circadian patterns in movement behavior (Cozzi et al., 2012; Davies et al., 2021). With these added movement terms, the full model for our mean response becomes

(1) $$\lambda_{\mathrm{ijt}}=\text{logit}\left(\alpha_{\mathrm{jt}}+\left(\beta_{h}*h\left(x_{\mathrm{tij}}\right)\right)+\left(\beta_{s}*S_{\mathrm{ijt}}\right)*\cos\left(\gamma_{\mathrm{ijt}-1}-\gamma_{\mathrm{ijt}}\right)+\beta_{l}*l_{\mathrm{ijt}}+\left(\beta_{d}*D_{\mathrm{ijt}}\right)*\ln\left(l_{\mathrm{ijt}}\right)\right)$$

where $h\left(x_{\mathrm{tij}}\right)$ represents a vector of spatial covariates for pack i at location j at time t. β represent the statistical coefficients associated with the relevant movement, habitat, and temporal covariates, denoted via subscript (i.e., $\beta_{s}$ is the statistical coefficient associated with season, S, for pack i and location j at time t). All movement modifiers ($l_{\mathrm{ijt}}$, $\ln\left(l_{\mathrm{ijt}}\right)$, $\cos\left(\gamma_{\mathrm{ijt}-1}–\gamma_{\mathrm{ijt}}\right)$) and their interaction terms were included as fixed effects, unlike the habitat selection covariates ($\beta_{h}*h\left(x_{\mathrm{tij}}\right)$) which were included as random effects.

### 2.5 Territorial covariates

To evaluate how territoriality influenced wild dog spatial behavior, we assessed how packs responded (selection or avoidance) to their previous space‐use (e.g., residency) as well as that of neighboring packs. For each given day spanning our movement dataset, we calculated the space use (utilization distribution, UD (Signer & Fieberg, 2021)) of all wild dog packs at four temporal scales, corresponding to the past week (7 days), 2 weeks (14 days), month (30 days), and 3 months (90 days). We then extracted each UD's 50% and 95% isopleth to generate distance‐to‐UD‐outline values ($\mathrm{dis}t_{x_{\mathrm{ow}n_{y}}}{\%}_{\mathrm{ijt}}$, where x is either 7, 14, 30, or 90 days, and y is either 50 or 95). We joined the space use data with the iSSF dataset, obtaining point‐specific (used and control point), spatiotemporal (two spatial scales and four temporal scales) territorial information for both own and neighboring territories (see Table S1). For these distance‐to‐UD calculations, points inside the isopleth boundaries were given a negative value, so that a value of 0 meant the used or control point was on the UD's outline. Lastly, for all points we determined the distances to all neighbors' co‐occurring GPS locations (within 3 h), and selected the distance to the nearest neighbor's location (Table S1; $\mathrm{dis}{t_{to_{\text{neigh}}}}_{\mathrm{ijt}}$). All territorial covariates (generically denoted in equation 2 as ${\mathrm{own}}_{\mathrm{tij}}$ or ${\text{neigh}}_{\mathrm{tij}}$) were standardized (mean‐centered and scaled by standard deviation). Because covariates required data with lags of up to 90 days, we concatenated our dataset such that we only included steps which had values for territory data at all timescales (i.e., 7, 14, 30, and 90 days) per *neighbor* and *own*. The addition of these territorial covariates resulted in the following model for our mean response lambda

(2) $$\;\lambda_{\mathrm{ijt}}=\text{logit}\left(\begin{array}{c} \alpha_{\mathrm{jt}}+\left(\beta_{h}*h\left(x_{\mathrm{tij}}\right)+\beta_{\mathrm{own}}*{\mathrm{own}}_{\mathrm{tij}}+\beta_{\text{neigh}}*{\text{neigh}}_{\mathrm{tij}}\right)+\\ \left(\beta_{s}*S_{\mathrm{ijt}}\right)*\cos\left(\gamma_{\mathrm{ij}-1}-\gamma_{\mathrm{ij}}\right)+\beta_{l}*l_{\mathrm{ijt}}+\left(\;\beta_{d}*D_{\mathrm{ijt}}\right)*\ln\left(l_{\mathrm{ijt}}\right) \end{array}\right)$$

where ${\mathrm{own}}_{\mathrm{tij}}$ represents covariates designating use of own territory (e.g., $\mathrm{dis}{t_{7_{\mathrm{ow}n_{95\%}}}}_{\mathrm{ijt}}$ or $\mathrm{dis}{t_{90_{\mathrm{ow}n_{50\%}}}}_{\mathrm{ijt}}$), while ${\text{neigh}}_{\mathrm{tij}}$ represents covariates designating neighbor territory use (e.g., $\mathrm{dis}{t_{to_{\text{neigh}}}}_{\mathrm{ijt}}$ or $\mathrm{dis}{t_{30_{\mathrm{ow}n_{50\%}}}}_{\mathrm{ijt}}$). The associated β values per territorial covariate represent the statistical coefficients for those terms. Territorial selection terms were included as random effects and were based on the end‐of‐step location. When territorial terms were tested as movement modifiers, they were based on the beginning‐of‐step location. See S1‐3 for more details.

### 2.6 Social covariates

To determine the influence of sociality on territoriality, we generated covariates for the number of adults (individuals >12 months old) in the focal pack (Table S1; $a_{\mathrm{ijt}})$, an indicator of pup presence (Table S1; where $p_{\mathrm{ijt}}=1$ if the pack had at least one individual <12 months old present and 0 otherwise) and an indicator of the pack's tenure (Table S1; $e_{\mathrm{jt}}$). Pack tenure, defined as the time since the formation of a pack, was calculated by subtracting the date the pack was first observed from the timestamp of the used or control point. Pack tenure had severely right‐skewed distribution, with a median of 2 years and a high of 7 years, which is consistent with the high turnover rate of packs (Woodroffe & Sillero‐Zubiri, 2020). We therefore made 2 years the cut‐off age to distinguish between “young” packs and “old” packs such that $e_{\mathrm{jt}}$ = 1 if time *t* is more than 2 years after pack *j* was first observed as a combination of male and female adults, and $e_{\mathrm{jt}}$ = 0 if time *t* is less than or equal to 2 years.

We also calculated neighboring pack information, such as neighboring pup presence (Table S1; where $\text{neig}{h_{p}}_{\mathrm{ijt}}=1$ if any neighboring pack had at least one pup present and 0 otherwise), and neighboring pack size (Table S1; $\text{neig}{h_{a}}_{\mathrm{ijt}}$). Designating neighboring pack size allowed us to calculate the difference between the focal pack's size and neighbors' pack sizes (Table S1; ${a_{\text{diff}}}_{\mathrm{ijt}}$) as well as the ratio between the focal pack and all animals in the area (Table S1; ${a_{\text{ratio}}}_{\mathrm{ijt}}$). Neighboring pack size, difference, and ratio values were aggregated to maximum, average, and minimum values when more than one neighboring pack was present (Table S1). The addition of these social covariates resulted in the following models for our mean response lambda

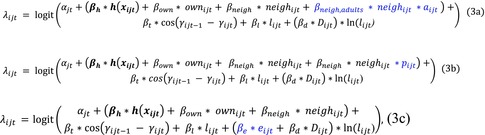

where the additional social covariates and their statistical coefficients are highlighted. Interaction terms between social modifiers and selection terms (or movement modifiers) were included as fixed effects. See S3 for more details.

### 2.7 Model selection

Due to the large number of covariates and predictions (Table 1), we used multiple steps in our model selection process. First, we wanted to find the core movement and resource terms relevant to wild dog habitat selection. We therefore fit a limited set of potential *base models* informed by previous work in our study area, and used model selection to select the highest performing one. Second, we sought to determine which spatiotemporal resolution of our territorial terms best predicted habitat selection of packs. We used model selection to pick the spatiotemporal combination of territorial covariates that improved model fit compared to the best performing *base model*. In the final component of our analysis, we compared a series of candidate models containing territorial and social information, as informed by the previous two steps, to test our predictions (see Table 1) on pack resource selection and movement behavior relative to territorial and social contexts of conspecifics. Our step‐wise approach avoids having to use model selection on one very large model that could lead to spurious results (Burnham & Anderson, 1998), and has been successful in other studies which (1) compare several spatiotemporal resolutions of landcover data (Nisi et al., 2022; Zeller et al., 2017) and/or (2) want to control for expected habitat use (e.g. finding a “base” model) while testing other ecological hypotheses (Davies et al., 2021). We used Bayesian informational criterion (BIC) to compare and select the best performing model at each stage. BIC has a stricter penalization process for increasingly complex models as compared to Akaike information criterion (AIC); AIC is prone to selecting overfitted models and therefore may not be as effective when comparing models with random effects (Link & Barker, 2006). See S3 for additional details.

**TABLE 1:** Process‐specific predictions and corresponding numbers.

Process	Prediction	Number
Territoriality	Packs will select for their own territorial cores at longer timeframes	PT1
Territoriality	Packs will select for their neighbors' boundaries (not their cores) at shorter timeframes	PT2
Territoriality	Packs will increase selection of scent marking‐friendly habitat (e.g. roads, pans) when in neighboring territory	PT3
Territoriality	Packs will move quicker in neighboring territories than in their own territories, as represented by a difference in step length distributions between own and neighboring territories	PT4
Sociality	Packs will seasonally vary in both their residency (a) and selection for neighbors' territories (b)	PS1a PS1b
Sociality	Larger packs will select for neighbors' territories, while smaller packs will avoid them	PS2
Sociality	Older packs will select for neighbors' territories, while younger packs will avoid them	PS3
Sociality	Packs without pups will select for neighbors' territories, while packs with pups will avoid them	PS4

*Note*: Each prediction is discussed in greater detail in Model Selection.

As described above, we first compared a limited set of models with habitat selection covariates and temporal movement modifiers to find our *base* model. Wild dog packs are known to move along roads (Abrahms et al., 2016), hunt in grassland and avoid mixed species woodland (Hofmann et al., 2023), avoid high human densities (O'Neill et al., 2020), and select rugged terrain, mopane woodland, and areas near pans for denning sites (Alting et al., 2021; Jackson et al., 2014). These previous studies informed a limited model set testing wild dog response to landcover data, seasonal changes in directional persistence (Pomilia et al., 2015), and daytime modification to the natural log of step length (Davies et al., 2021). Due to the increasing model complexity later on, we limited our candidate set in this first stage to models with no more than two random effects (i.e., two landcover data selection coefficients).

Second, to determine at which spatiotemporal scale territorial selection operates, we split our candidate model sets into *own* and *neighbor* models. Specifically, *own* models had one additional territorial selection term as compared to the *base* model denoting a pack's residency, and *neighbor* models had one additional selection term denoting neighbors' space use (Table S1 for all *own* and *neighbor* terms). We included the top‐performing base model from the previous stage in both candidate model sets (*own* and *neighbor*) to test whether the additional territorial covariates were improving model performance. We predicted that wild dog packs would select for their own territory core (PT1) and select their neighbors' boundaries. We predicted selection for neighbors' boundaries given the high overlap between packs as predicted by the IPH, and the need to both scent mark and gather information (via scent marks) on neighbors (PT2). We also predicted that residency would operate at a greater temporal scale than selection for their neighbors' territory given focal pack preferences and familiarity (i.e., selecting for own core at the 90 day scale as compared to the 14 day scale, but selecting neighboring boundaries at the 14 day scale as compared to the 90 day scale; PT1 & PT2). We used model selection in each set to find the best performing model for *own* and *neighbor* models (see S4), respectively, which revealed the temporal (7, 14, 30, or 90 days) and spatial (distance to boundary or core) scales at which territorial selection occurs.

Once we had determined the relevant territorial terms, we ran two global model selection procedures to determine (1) how territoriality influenced habitat selection (PT3) and movement (PT4) and (2) whether inter/intra‐pack social dynamics influenced territoriality (PS1–PS4). In our *global territorial* model set, we predicted that the best performing model would include terms for both residency and neighboring space use. We therefore took our highest performing *base* model, added one of each territorial selection term (i.e., the *own* term from the best performing *own* model and the *neighbor* territorial term from the best performing *neighbor* model), and tested additional interaction terms between movement terms or landcover features, with each territorial term, to find a final *global territorial* model. Specifically, we predicted that selection for features which facilitate scent marking behavior, such as roads and pans (see Claase et al., 2022), would change when in neighboring territories (PT3). We hypothesized packs would select for features relevant to scent marking behaviors when in neighboring territories as they should be useful locations for gathering information on neighboring packs. We also predicted packs would move faster in overlap areas with greater neighbor presence (PT4), and so included models with interactions between each territorial selection term and either directional persistence or the natural log of step length. In these movement‐modification interactions, we included the territorial information at the beginning of the step. Additionally, we predicted stronger selection for roads when in neighboring territory to facilitate faster movement and therefore avoidance of potential confrontation with neighbors (PT3) (Abrahms et al., 2016).

Fourth and finally, to investigate whether inter/intra‐pack social dynamics influenced territoriality, we created a *global social* model set to test whether and how social terms which interacted with territorial terms improved model fit, as compared to the *global territorial* model. We predicted that selection of neighboring territory would be modified by (1) seasonality, (2) pack size, (3) pack tenure, and (4) pup presence. We predicted that seasonality would alter selection of territorial space given seasonal changes in home range sizes (Pomilia et al., 2015). In this context, we predicted increased residency (PS1a) in the pre‐denning season due to the importance of den site selection to pup survival (Alting et al., 2021), and increased selection of neighboring territory (PS1b) in the post‐denning season due to territory enlargement following the more spatially‐constricted denning period. We predicted that larger packs (who have more mouths to feed), packs without pups (who are not limited by pups' limited mobility), and older packs (which have more experience) would have increased selection for neighboring pack territory compared to smaller packs (PS2), younger packs (PS3), and those with pups (PS4), respectively. Given mortality risks from direct confrontation between packs (Jordan et al., 2017), we predicted that the degree of a pack's residency should be inverse with their ability to safely investigate their neighbors. To test these predictions, we compared models that contained interaction terms among territorial selection terms or movement modifiers and social information in our *global social* candidate model set. In this model set, we took the highest performing *global territorial* model and added an additional interaction between a territorial term and social data (e.g., neighbor's boundary and pack tenure) or included a movement modification interaction (e.g., neighbor's boundary and step length). We identified our candidate models by social information (*pack size*, *pup presence*, *pack tenure*, and *seasonality*) to determine whether each social component impacted territorial selection (either *own* or *neighbor* selection terms), or movement (either the natural log of step length or directional persistence) accordingly. We compared all *global social* models to the highest performing *global territorial* model to determine whether and how social interaction terms improved model fit. We ranked these *global social* models to determine which outperformed the *global territorial* model, even if the model was not the highest performing one in the set.

We calculated the relative selection strength (RSS) (Avgar et al., 2017) for each variable retained in the top‐performing *global territorial* model and the highest performing models in each social category of the *global social* model set (*pack size*, *pup presence*, *pack tenure*, and *seasonality*), only if it outperformed the *global territorial* model. We used the RSS to determine how habitat or social covariates of interest influence movement and selection of territorial space in wild dog packs. The beta values from our movement modifier covariates in our *global territorial* model were used to update the parameters for both step length and turning angle distributions of wild dog packs in the absence of resource selection (Avgar et al., 2016). We used these parameters to generate temporally modified step length and turning angle distributions of wild dog packs where relevant.

## 3 RESULTS

**FIGURE 1:**
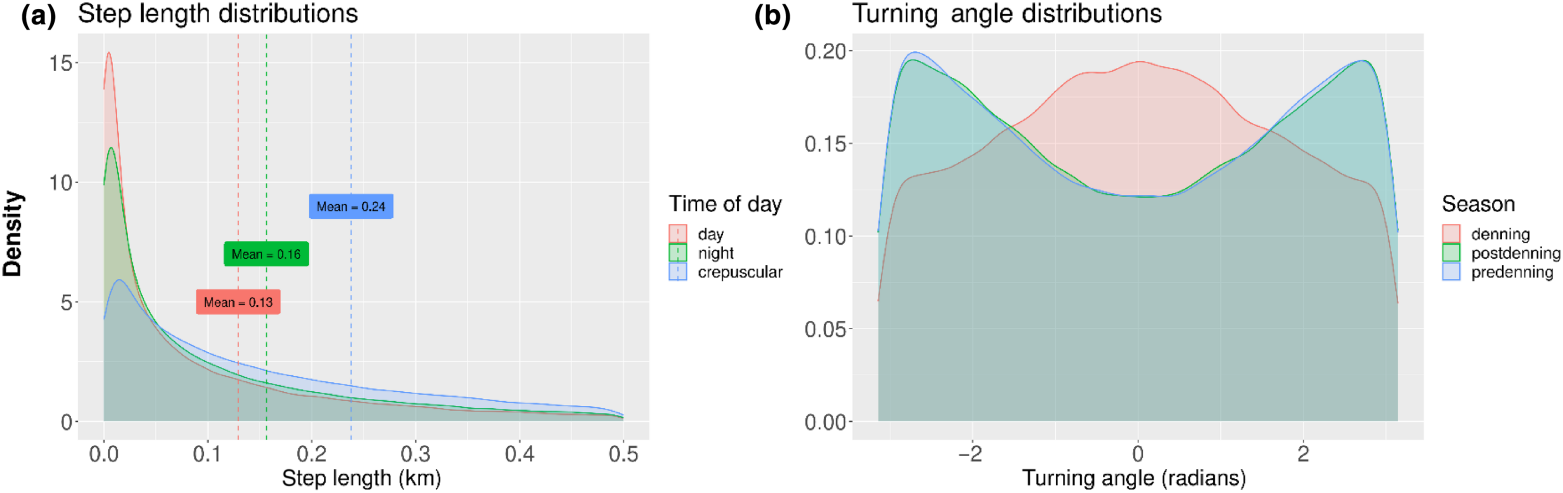
African wild dogs are more active during crepuscular hours than nighttime or daytime, and have increased recursiveness during the denning season. (a) Density plot of step length distributions in kilometers specific to each time of day, with each distribution's mean shown in dashed lines and labeled accordingly. (b) Density plot of turning angle distributions in radians updated with seasonal modifiers.

Wild dog packs select for grassland and roads, and their movement is influenced by both time of day and season. Specifically, our best performing *base* model showed selection for grassland, a time of day modifier to the natural log of step length suggesting packs move more during crepuscular hours than at day or night (Figure 1a), and a seasonal modifier to turning angle suggesting packs had greater recursive movement during the denning season (Figure 1b).

Wild dog movement and resource selection is influenced by the spatial layouts of both their territory and their neighbors'. Comparing model performance among *own* and *neighbor* territorial models revealed the spatiotemporal scales for territorial selection. Specifically, wild dog packs select for proximity to neighbors' 30‐day boundary (95% isopleths; Table S2), and proximity to their own 90‐day core (50% isopleths; Table S1), supporting our prediction that wild dog packs respond to their neighbors' space use and their own at different spatiotemporal scales.

**FIGURE 2:**
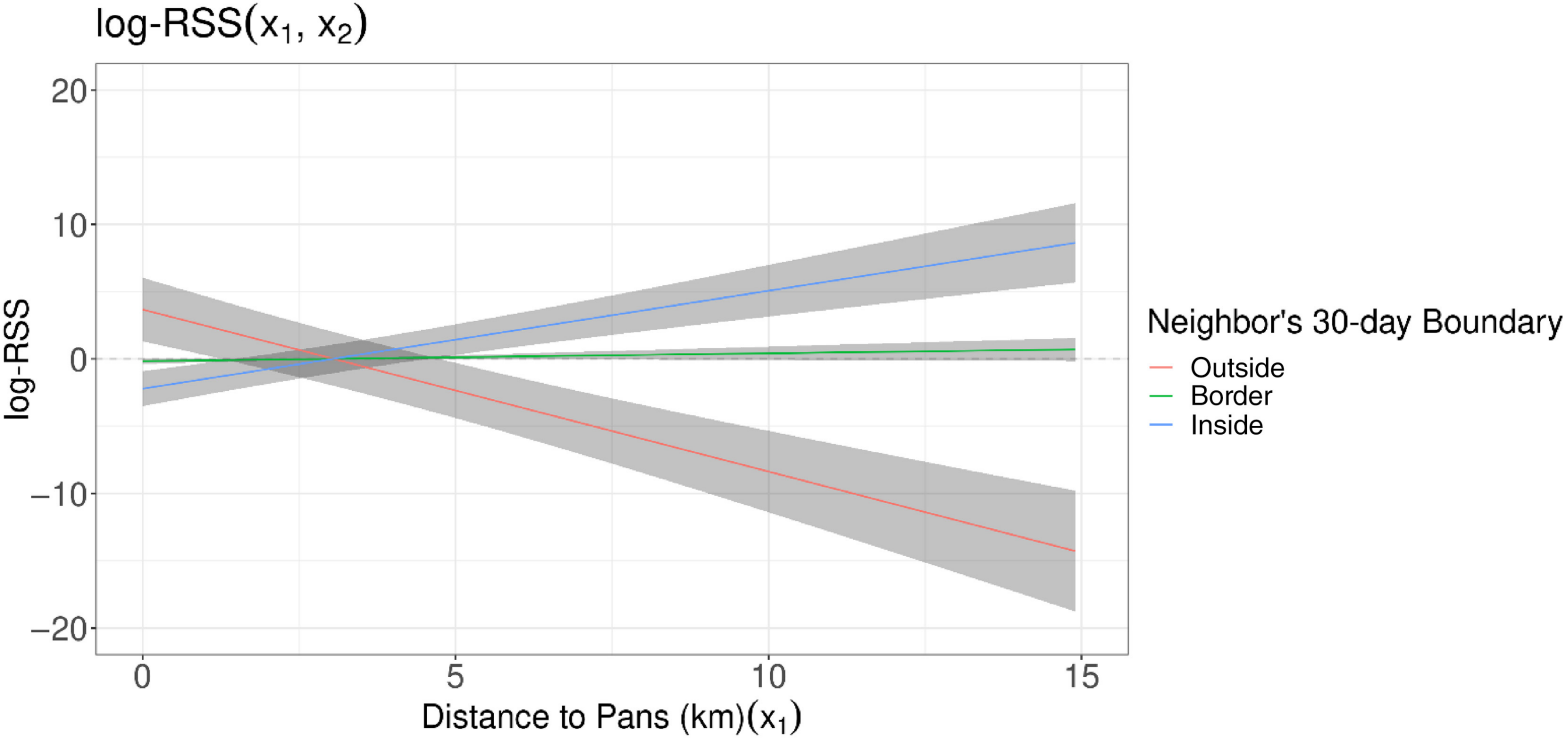
African wild dog packs mediate selection for pans when inside their neighbors' territory. Here the log relative selection strength (log RSS) for the interaction between distance to pans and distance to neighbor's 30 day boundary is shown at any x_1_ position (which is, any distance from pan value shown on the x‐axis) relative to position x_2_, the standardized mean distance from pans (where x_2_ = 0.0055). The log RSS was estimated at three different levels of scaled distance to neighbor's 30 day boundary: outside (the maximum distance, shown in red), border (at 0 km, the mean distance, shown in green), or inside (the minimum distance, shown in blue), and then we unscaled our x‐values to show the log RSS on the scale of kilometers. Each log RSS line is outlined in gray by 95% error bars.

In our best performing *global territorial* model, packs strongly selected for proximity to neighbors' 30‐day boundary (95% isopleths; $\beta_{\text{neigh}}=-9.84\pm 0.16$) with some selection for their own 90‐day core (50% isopleths; $\beta_{\mathrm{own}}=-0.26\pm 0.15$). Per our prediction, wild dog packs mediate habitat selection when in neighboring territories. This best performing model included an interaction term between distance to pans and distance to neighbor's boundary ($\beta_{\text{neigh}:\mathrm{pan}}=-0.611\pm 0.09276$; Figure 2) which was statistically significant, suggesting that packs are more likely to avoid pans when farther inside their neighbor's territory (Table S3). See Table 2 for a full list of all covariates and interaction terms included in our best performing *global territorial* model (Table 2).

**TABLE 2:** All covariates included in the top performing *global territorial* model, their estimates, and standard errors.

Term	Estimate	Std. error
Distance to neigh 30‐day bound.	−9.84	0.17
Distance to own 90‐day core	−0.27	0.16
Distance to pans	0.30	0.12
Distance to grassland	0.03	0.05
Distance to roads	0.18	0.13
Step length (km)	−0.77	0.02
Dist. to neigh: dist. to pans	−0.61	0.09
ln(step length): daytime	0.07	0.02
ln(step length): nighttime	0.15	0.01
ln(step length): crepuscular	0.40	0.02
cos(turning angle): denning	−0.23	0.05
cos(turning angle): postdenning	0.23	0.06
cos(turning angle): predenning	0.22	0.05

**FIGURE 3:**
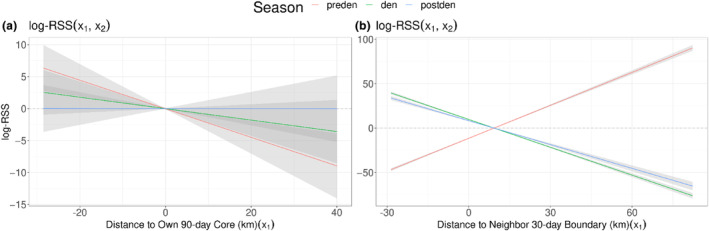
Packs mediate the strength of residency as well as selection for neighbors' territory depending on the denning season. Here the log RSS is shown at any position x_1_ (any position along the x‐axis) relative to the position x_2_, which is the mean scaled distance from a pack's (a) own 90‐day core (x_2_ = 0.006) and (b) neighbors' 30‐day boundary (x_2_ = 0.16). We compare selection at position x_1_ relative to selection for the overall mean distance to a territorial boundary for each season: predenning, denning, and postdenning. We unscaled our x‐values to show the log RSS on the scale of kilometers.

Season, pup presence, pack tenure, and pack size all mediated pack response to their neighbors. At least one model per social interaction type (seasonality, pup presence, pack tenure, and pack size) outperformed the highest performing *global territorial* model (Table S4). The top performing *global social* model included seasonal interactions with both *own* and *neighbor* territorial selection terms (Table S4). Per our prediction, the strength of selection for proximity to a pack's own core varied across seasons, with stronger selection in the pre‐denning season and no selection in the post‐denning season (Figure 3a; Table S5). Packs changed selection of neighboring boundaries across seasons (Figure 3b; Table S5), avoiding their neighbors in the predenning season while selecting for them in the other two.

**FIGURE 4:**
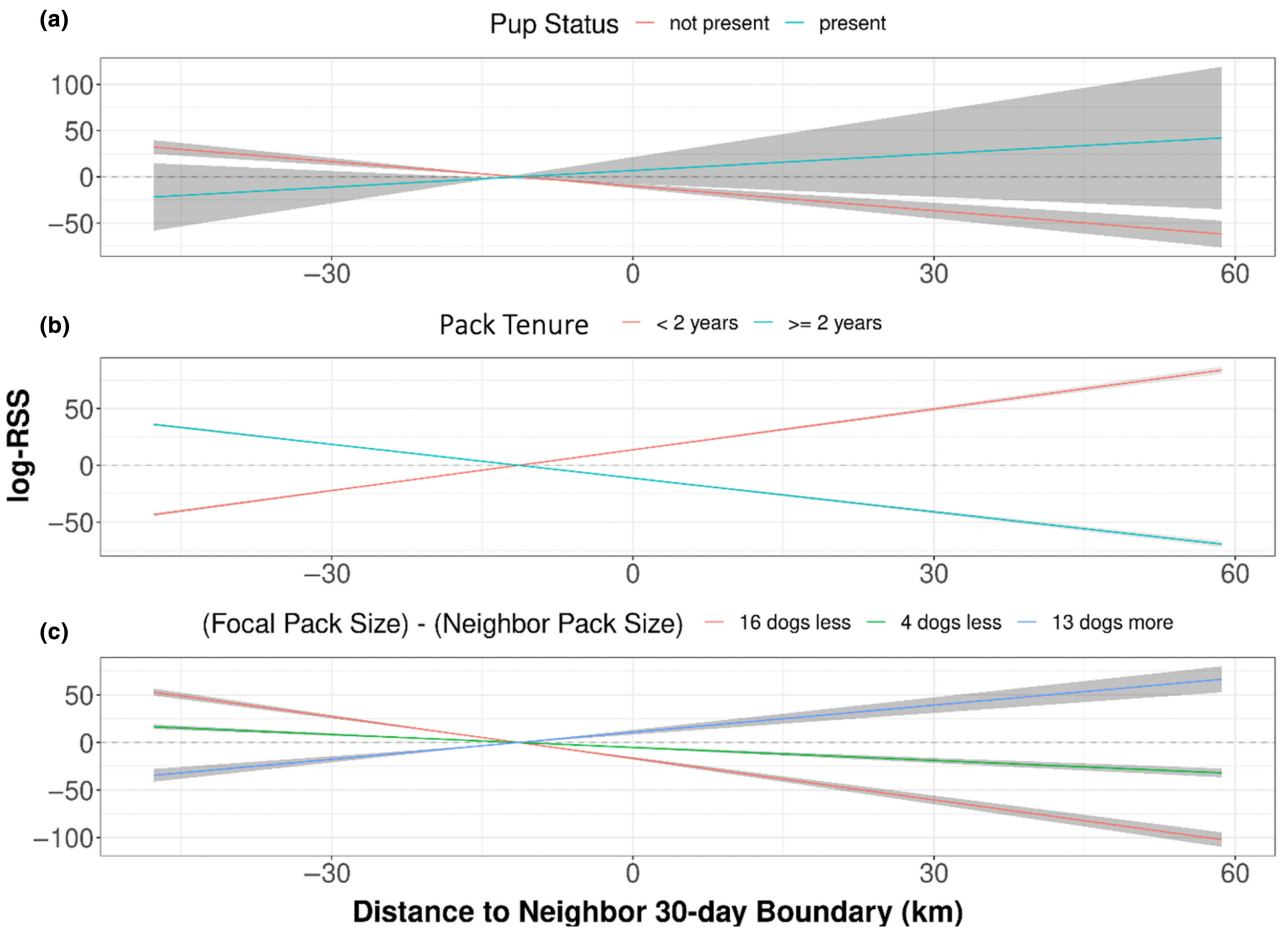
The social composition of packs determines whether packs select for or avoid their neighbors. Here we show the log RSS for the distance to a pack's neighbors' 30‐day boundary (position x_1_) relative to the standardized mean distance (position x_2_, where x_2_ = 0.16) depending on (a) pup status (either present or not present), (b) pack tenure (either less than 2 years or greater than or equal to 2 years), and (c) difference in pack size between focal and neighboring pack (the greatest positive difference, the mean, and greatest negative difference). We unscaled our x‐values to show the log RSS on the scale of kilometers. Positive log RSS at small distances to neighboring boundary suggests selection for locations close to or within neighboring boundaries, while a negative log RSS suggests avoidance.

While other models including interactions between pup presence, pack tenure, and pack size with territorial terms were not the highest performing *global social* models, we still report their results here. By interacting neighboring territory with any one of the social covariates (but not own territory or movement behavior), the model outperformed the *global territorial* model. For instance, when pups were present in the focal pack, packs showed avoidance of neighboring boundaries and selection when pups were not present (Figure 4a; Table S5). We also found that older packs would select for their neighbors' boundaries while younger packs would avoid them (Figure 4b; Table S5). We found no evidence that pup presence nor pack tenure significantly influenced movement (Table S4). Additionally, packs selected for territories of packs that were relatively smaller than them and avoided the territories of larger neighbors (Figure 4c; Table S5). Indeed, the effect sizes of social‐territorial interaction terms were significantly larger than the habitat‐territorial interaction term in all cases.

## 4 DISCUSSION

Competition influences the space use of individuals (Sells & Mitchell, 2020). Here, we show that competition among conspecifics influences wild dog resource selection across territorial space. Wild dog sociality, specifically pack tenure, pack size, pup presence, and seasonal breeding behaviors, all mediate how packs react to neighboring conspecifics, and seasonality mediates pack residency. We used an integrated step selection function (iSSF) to illuminate territorial interactions between wild dog packs, such as the temporal scale at which they respond to neighboring space use, how territoriality mediates resource selection, and social influences on territorial processes. These findings have critical implications for our understanding of the influence of competitive interference on resource selection, and hint at the inter‐ and intrapack factors which mediate competition.

Wild dogs actively select for proximity to their neighbors' boundary in some contexts but not others. Previous work has shown that wild dog packs have extremely few direct encounters (Jordan et al., 2017) despite large overlap (Pomilia et al., 2015). Altered habitat selection, as demonstrated by the difference in selection for pans when inside versus outside neighboring boundaries, may be one of the mechanisms packs use to move in overlapping areas while avoiding neighbors. Pans are used by wild dogs to scent mark (Parker, 2010) and, given their avoidance of lion‐heavy area such as floodplains near riverbanks (Cozzi et al., 2013), could also be important seasonal water sources. In this situation, packs may avoid habitat they otherwise prefer because the risk of competitive interference from neighbors reduces habitat quality in overlap areas (O'Neil et al., 2020). It is also possible that analyzing movement data at finer scales could reveal selection of other habitat features and or movement behaviors which are also mediated by territorial space, as animals have temporal grain‐dependent resource selection and movement (Nisi et al., 2022).

While inter‐pack encounters occur infrequently in African wild dogs (Jordan et al., 2017) they can be fatal in this (Creel et al., 2002) and other species (King, 1973), especially where competing groups vary in size (Wrangham & Glowacki, 2012). Avoiding larger groups of conspecifics may therefore minimize fatality risk (Creel et al., 2002). Here we found that wild dogs increased selection of neighboring territory when the neighboring pack was smaller than their own and avoided territories of larger packs. In general, carnivores have restricted habitat selection when the risk is greater: the risk of pup mortalities has been shown to affect wolf territory size and conspecific interactions (Smith et al., 2015), and anthropogenic disturbance has a much greater effect on selection for den sites and scrape sites in pumas than “everyday” habitat selection (Wilmers et al., 2013). Given the risk of a mortality event from an encounter, it follows that smaller packs (less available fighting adults), packs with vulnerable young pups, and younger packs (with less social cohesion and experience), all showed strong avoidance of their neighbors.

The seasonal effect on selection for own core and neighbor boundary is similar to behaviors found in other species, where seasonal movements of prey (Brandell et al., 2020) or reproductive needs (Johansson et al., 2018) may cause territorial carnivores to alter their spatial organization. Wild dog packs breed so that pups are whelped close to the coldest day of the year (Woodroffe et al., 2017). This temperature restriction results in consistent seasonal influences on space use and a consistent denning season (Pomilia et al., 2015). Den site selection is an important process for wild dogs to avoid both conspecifics and lions (Alting et al., 2021; Davies et al., 2016), which could explain why wild dogs showed stronger selection for their own territories (and simultaneous avoidance of their neighbors') in the predenning season, which is when they must find and choose a den site. Given that wild dog ranges shrink considerably in the denning season, packs have weaker residency once packs leave the den (postdenning season) and reclaim area beyond the limited denning‐season range.

The time required for territorial maintenance is often the most important cost determining the evolutionary stable strategy of territoriality in both empirical and theoretical studies (Ord, 2021; Sells & Mitchell, 2020; Varga et al., 2020). Our results support the idea that wild dog packs may dually seek to monitor neighbor presence while avoiding physical encounters given the strong selection for neighbors' boundaries (and not cores) at an intermediate time scale (and not the nearest contemporaneously occurring neighbor; Table S1). Wild dog packs do use longer time scales, however, when selecting space within their own territories (90 days). Given that available data limited our temporal aggregation of residential and neighbors' space use to the past 3 months (90 days), packs may structure residency on an even longer time frame.

The results of our base model are consistent with previous publications from the study area, which report selection for roads (Abrahms et al., 2016), grassland as primary hunting grounds (Alting et al., 2021), crepuscular activity patterns (Cozzi et al., 2012; Davies et al., 2021), and seasonal changes in territory use due to breeding status (Pomilia et al., 2015). In the denning season, packs move with increasing tortuosity, reflecting the frequent movement recursions from and towards their den site (Berger‐Tal & Bar‐David, 2015), which corresponds with a smaller territory size (Pomilia et al., 2015). We also found that wild dogs increased movement speed during crepuscular hours as compared to day time (Figure 1a) (Davies et al., 2021), which is consistent with heat restrictions on hunting capacity of wild dogs (Woodroffe et al., 2017). Environmental features are much less impactful on habitat selection as compared to territorial information (see the much smaller effect sizes for environmental covariates with respect to territorial covariates in Table 2). While adding interactions between territorial terms and environmental features is beyond the statistical scope of this paper, our second highest performing *global territorial* model included an interaction between grassland and neighboring territory (Table S3). Other research suggests that conspecifics have a much greater impact on habitat selection than what has been accounted for, specifically through density dependence mechanisms (Avgar et al., 2020; Smith et al., 2022). Accounting for dynamics such as experience, composition, or breeding status of conspecifics is equally important in understanding habitat selection and movement processes. Indeed, the effect sizes of social‐territorial interaction terms exceeded those of habitat‐territorial interaction terms.

Our study suggests that sociality mediates territorial processes in wild dog packs, as we see multiple social‐based interactions cause selection to change to avoidance within neighbors' territorial space. These results join the growing body of literature (Matthiopoulos et al., 2019; Smith et al., 2022) which argue that the predictive capacity and understanding of animal space use will be limited if we fail to consider the density dependent processes, particularly for group living species whose space use is strongly affected by social structure (Webber et al., 2022). However, unraveling the mechanisms underneath conspecific competition in carnivores extends beyond the predictive capacity of space use; the sociality and cultural components of group living species are now being recognized as integral to conservation management objectives (Brakes et al., 2021; Goldenberg et al., 2019; Maldonado‐Chaparro & Chaverri, 2021). African wild dogs are the most endangered carnivore in Southern Africa, and habitat fragmentation resulting in increased human–wildlife conflict is the most severe threat to population stability (Woodroffe & Sillero‐Zubiri, 2020). Experiments have demonstrated the capacity to utilize conspecific scent to alter space use and or movement of carnivores (Arnold et al., 2011; Christensen & Radford, 2018; Jackson et al., 2012; Sliwa & Richardson, 1998), leading the BPC team to test the production of a synthetic “BioBoundary” (Apps et al., 2013). To predict responses of groups or individuals to “invasions” or “new neighbors” (i.e., a BioBoundary deployment pattern meant to mimic residency in a conflict‐prone area, such as areas with livestock), models which incorporate the differences in movement or selection between groups will be critical in managing packs with varying social compositions. Our model suggests, for example, that older and larger packs may not avoid a location surrounded by a BioBoundary but a smaller, younger pack could. Therefore, the BioBoundary may have a role in dissuading these reproductively vulnerable (and therefore less competitive) packs from settling in potential human‐conflict areas. Human–carnivore conflict mitigation tools are often utilized without proper experimentation (van Eeden et al., 2018); studies such as ours can inform strategic development and more targeted testing of potential conflict tools for other territorial species, which is becoming increasingly relevant for species such as carnivores which are prone to human‐conflict and threatened by extinction (Johnson et al., 2023; Woodroffe & Sillero‐Zubiri, 2020).

## AUTHOR CONTRIBUTIONS

**K. Whitney Hansen:** Conceptualization (lead); data curation (lead); formal analysis (lead); investigation (lead); methodology (lead); project administration (lead); validation (lead); visualization (lead); writing – original draft (lead); writing – review and editing (lead). **Nathan Ranc:** Methodology (supporting); validation (supporting); writing – review and editing (supporting). **John Morgan:** Formal analysis (supporting); methodology (supporting); writing – review and editing (supporting). **Neil R. Jordan:** Supervision (supporting); writing – review and editing (equal). **J. Weldon McNutt:** Funding acquisition (equal); writing – review and editing (supporting). **Alan Wilson:** Funding acquisition (lead); writing – review and editing (supporting). **Christopher C. Wilmers:** Conceptualization (supporting); formal analysis (supporting); investigation (supporting); methodology (supporting); supervision (lead); writing – review and editing (lead).

## CONFLICT OF INTEREST STATEMENT

The authors have no conflicts of interests relating to this research.

## Supporting information


Data S1.

## Data Availability

Due to the endangered status of our studied species, data is not made publicly available. However, data contributors make the data available under reasonable request. Please contact JM, NJ, and AW for more information.
