# Supplementary material for: How territoriality and sociality influence the habitat selection and movements of a large carnivore
Source: Ecol Evol. 2024 Apr 16;14(4):e11217. doi: 10.1002/ece3.11217 (PMC11019303; doi:10.1002/ece3.11217)
Supplement: Supplementary file 1 — Data S1. [file ECE3-14-e11217-s001.docx]

SUPPLEMENTAL INFORMATION

| **Equation term** | **Covariate** | **Description** |
| --- | --- | --- |
| ${own}_{ijt}$ | ${dist\_x\_own\_y\%}_{ijt}$  ${UDx\_own}_{ijt}$ | Distance to own y = 50% or y = 95% isopleth border, extracted from an x-day UD (x = 7, 14, 30, or 90).  Value of own x-day UD (x = 7, 14, 30, or 90). |
| ${neigh}_{ijt}$ | ${dist\_x\_neigh\_y\%\_avg}_{ijt}$  ${dist\_x\_neigh\_y\%\_min}_{ijt}$  ${UDx\_neigh\_avg}_{ijt}$ ${UDx\_neigh\_max}_{ijt}$  ${dist\_to\_neigh}_{ijt}$ | Average or minimum distance value to neighbors’ y = 50% or y = 95% isopleth borders. Borders extracted from an x-day UD per neighbor (x = 7, 14, 30, or 90).  Average or maximum value of neighbors’ x-day UD’s (x = 7, 14, 30, or 90).  Distance to closest neighboring pack within 3 hours. |
| $p_{ijt}$ | $p_{ijt}$  ${p\_neigh}_{ijt}$ | Binary covariate denoting whether focal pack has pups.  Binary covariate denoting whether any neighboring pack has pups. |
| $e_{ijt}$ | $e_{ijt}$ | Binary covariate denoting whether pack is less than 2 years old (0) or 2 years or older (1). |
| $a_{ijt}$ | $a_{ijt}$  ${a\_neigh\_max}_{ijt}$  ${a\_neigh\_min}_{ijt}$  ${a\_neigh\_avg}_{ijt}$  ${a\_diff\_max}_{ijt}$ ${a\_diff\_min}_{ijt}$ ${a\_diff\_avg}_{ijt}$  ${a\_ratio\_max}_{ijt}$ ${a\_ratio\_min}_{ijt}$ ${a\_ratio\_avg}_{ijt}$ | Number of adults in focal pack.  Max, min, and average number of adults in neighboring packs.  Focal packs’ number of adults minus the max, min, or average number of adults in neighboring packs.  Focal packs’ number of adults divided by the total number of individuals on the landscape (focal pack adults + neighboring packs’ adults) |

**S1: Summary Table**

The Summary Table contains a description of all territorial and social covariates tested in the model selection process. Here we list the associated umbrella term used in equations 1, 2, and 3a-c, each possible covariate calculated to approximate the umbrella term, and a description of each covariate. Each territorial covariate was included as a linear term or as a linear term plus in an interaction term with a habitat feature, movement modifier, or social covariate. Social covariates were only included in interaction terms with movement modifiers or territorial covariates. Model selection procedures were used to 1) test how territorial and social information improved model performance, and 2) test which proxies for territorial (e.g.. which spatiotemporal scale) or social information (e.g. which quantification of pack size) best explained resource selection and movement of wild dogs. The Covariate column represents all possible proxies that were tested in model selection procedures, and the Description column contains information on how they were calculated.

**S2: Territorial Covariates**

In addition to quantifying territorial space use as “distance-to-UD-border”, we also quantified territory use as a UD value per pixel for both *own* and *neighbor* (table S1). We aggregated these neighbor UD values when more than one pack was present as an average of all values and as “maximum” UD value to generate two distinct terms (table S1). These were included in the model testing process against the “distance to isopleth” values, but were not in the highest ranking models explaining selection for neighbor or own territory. Likewise, we aggregated the *neighbor* distance-to-UD-border values in two ways: first as an average of all values (so all packs which had data were included in the average) and second as the “closest distance” (i.e. whichever pack was closest) to generate two distinct terms (table S1). Notably, when testing spatiotemporal combinations of pack response to own and neighbor space use, we put a cap on space use at 90 days because most animals did not have more than 90 days of cumulative data.

**S3: Model Selection Procedures**

**Figure S3:**

**
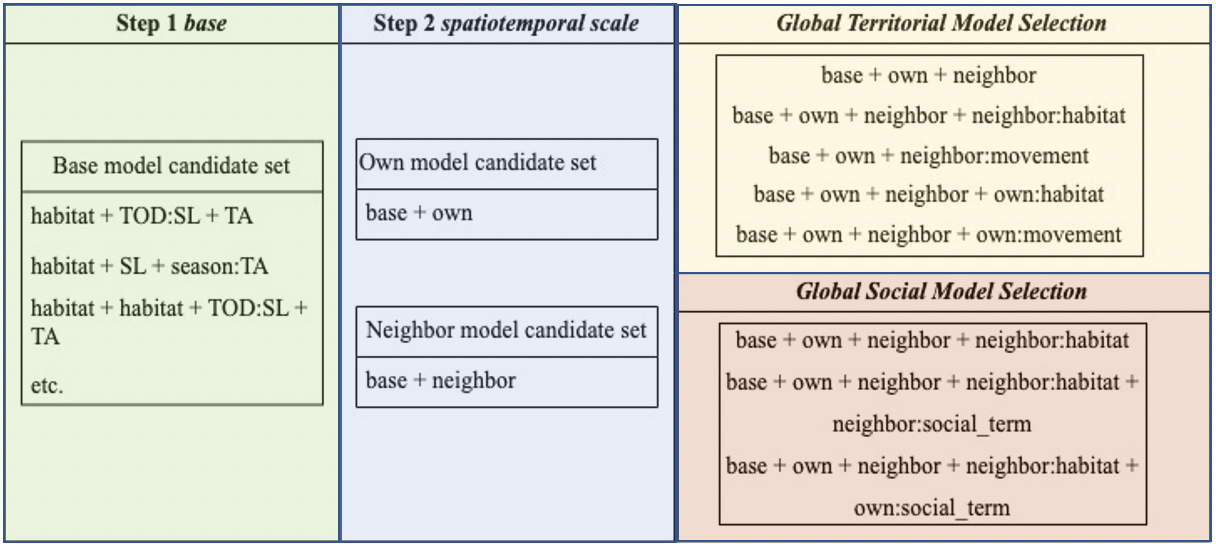
**

A conceptual diagram of model selection steps executed in this study. In the first step our goal was to determine the core movement and habitat selection terms describing wild dog pack resource selection. Habitat data include the distance to each landcover feature listed in the manuscript, including floodplain, mopane, mixed species woodland, grassland, pans, and roads. Temporal movement modifiers to either step length (SL) or cosine of the turning angle (TA) include time of day (TOD) and season. In the second step we used model selection to narrow down the spatiotemporal scale of territorial terms which best influenced habitat selection of packs. Accordingly, the *own* and *neighbor* terms refer to the equation terms from table S1, and therefore represent any possible spatiotemporal scale of territorial terms as described in table S1. The Global Territorial Model Selection box describes one of our two principal model selection procedures where we test our predictions on how territoriality influences movement behavior and habitat selection of packs. The Global Social Model Selection box designates the second principal model selection procedure, where we test our social-driven predictions. The “social_term” refers to the equation terms *p*, *e*, and *a* from table S1, and therefore represent any possible type of social covariate as described in table S1. The “movement” terms refer to either step length or cosine of the turning angle.

The overparameterization of models in the *global territorial* and *global social* model selection procedures led to certain territorial random effect terms having non-finite standard deviations, so that some models contained non-positive definite Hessians (meaning there were nonpositive eigenvalues in our correlation matrix). Very small negative numbers in the correlation matrix can occur due to rounding or noise in the data, and in our case were due to very small random effect variances. When included, these random effects explained close to no variance when the covariate was included 3 or more times in the model. We therefore excluded random effects of terms that were represented in multiple interactions, so that any given model contained a maximum of 3 random effects (two habitat selection covariates, and one of the territorial selection covariates depending on which was being tested in different interaction terms).

Given the more limited social data which was required for testing hypotheses in this final stage (we had to concatenate our dataset further to include only those points that had demographic data associated with GPS points), we reran the *global territorial* models on the limited dataset to find the top performing model. We used this highest performing model as a comparison to the models in the *global social* model candidate set in order to test whether the additional social covariates (see Table S1) improved model performance on the limited dataset as well.

**S4: Model Ranking Tables (*Own*, *Neighbor*, *Combination*, and *Social*)**

**
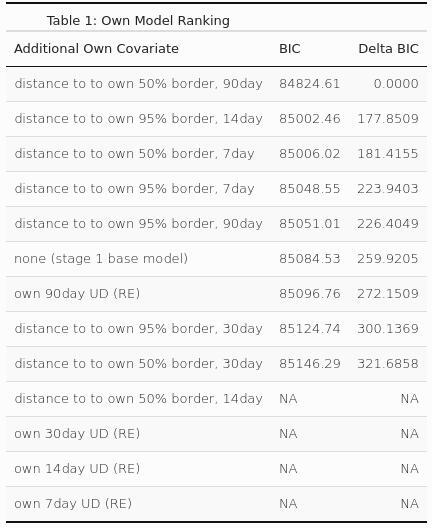

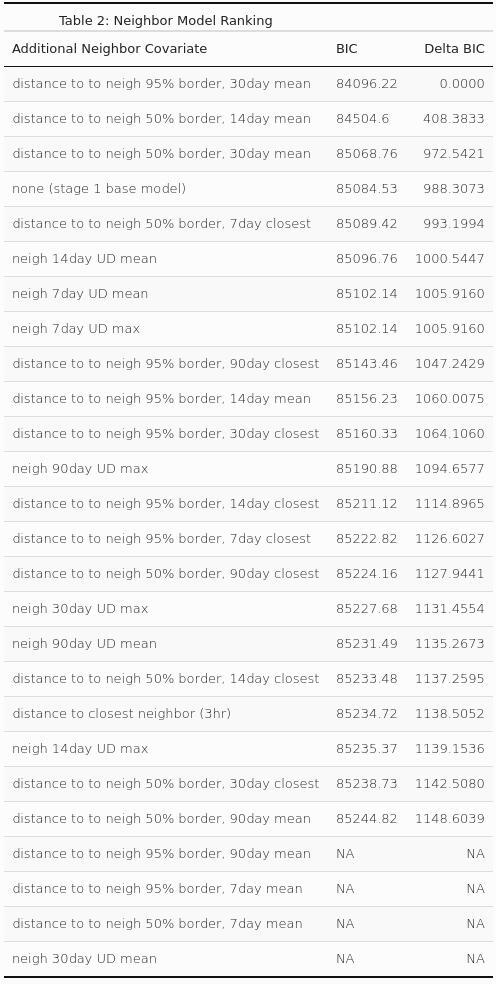
**

Tables 1 and 2 depict the rankings of *own* and *neighbor* models, respectively (see *Step 2* in Figure S3). These tables are the results of our model selection procedure to determine the optimal spatiotemporal approximation for residency and neighboring territory influence on habitat selection of wild dog packs. In the “Additional Covariate” column we describe the territorial covariate added to the highest performing *base* model (from stage 1), and we rank models by BIC score. The row with the Delta BIC score of 0 shows the territorial covariate used in the remaining model stages.

**
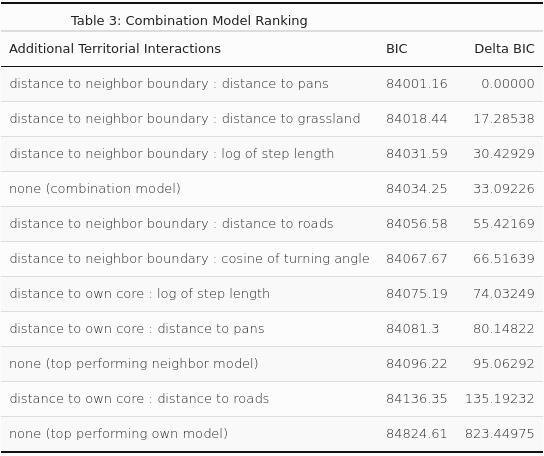
**

Table 3 ranks the highest performing models from our *global territorial* model selection procedure. In this table we have included the top performing *own* and *neighbor* models for comparison. The *combination* model is a base model plus the top performing *own* territorial term and the top performing *neighbor* territorial term (see Figure S3, *Global Territorial Model Selection*). The remaining models in this table include one more additional interaction term, which are listed in the “additional territorial interactions” column. These models are ranked by BIC score, and show that the top performing models do not only account for territorial selection but also interactions between territorial space and habitat use.

**
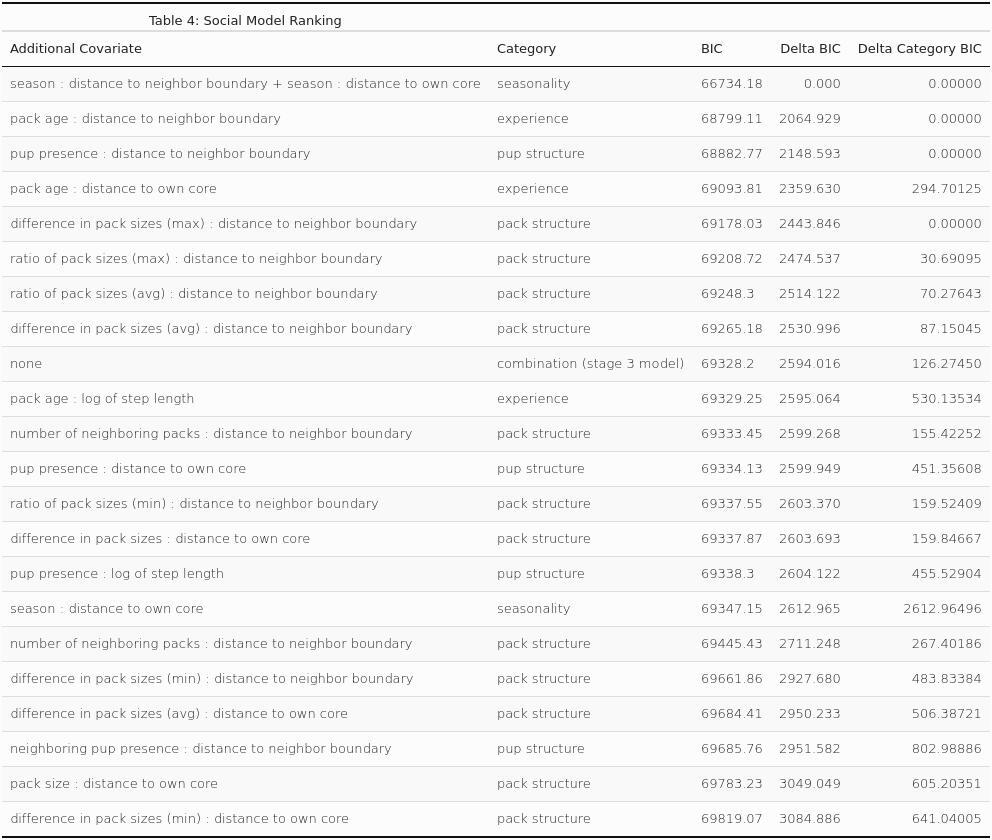
**Table 4 ranks the highest performing models from our *global social* model selection. In this table we have included the top performing *global territorial* model, which was re-run on a more limited dataset (see S3). Each row describes the additional covariate, which is a territorial term interacting with a social component (a proxy for either season, pack size, pack age, or pup presence; see Table S1), the social label of the model, overall BIC score, overall delta BIC, and a category-specific delta BIC. The social label of the model was merely used as a descriptor to keep track of which social information was or wasn’t important in mediating territorial habitat selection. These models are ranked by overall BIC score. This table demonstrates that additional social information, which include any of the possible categories we delineated above (Table S1), improves model fit.

S5: Social-territorial interaction covariates across top *global social* models


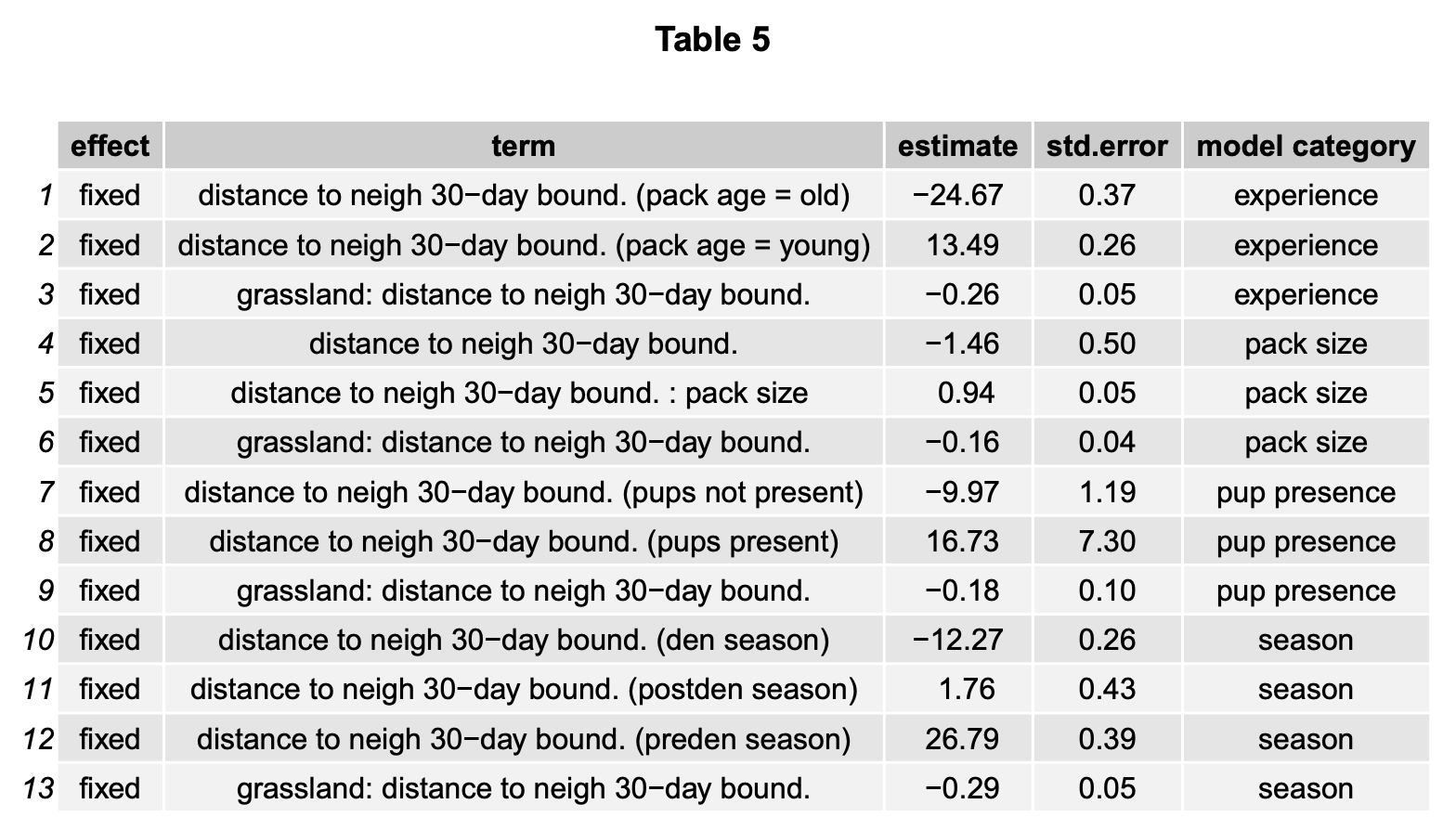


Table 5 compares the territorial and social-territorial interaction terms across the four top performing *global social* models referred to in the main text. We include the estimate, standard error, and a descriptive column called ‘model category’ which designates which social model the term belongs to. All terms with the ‘experience’ model category, for instance, were in the same model.

S6: Characteristics of packs


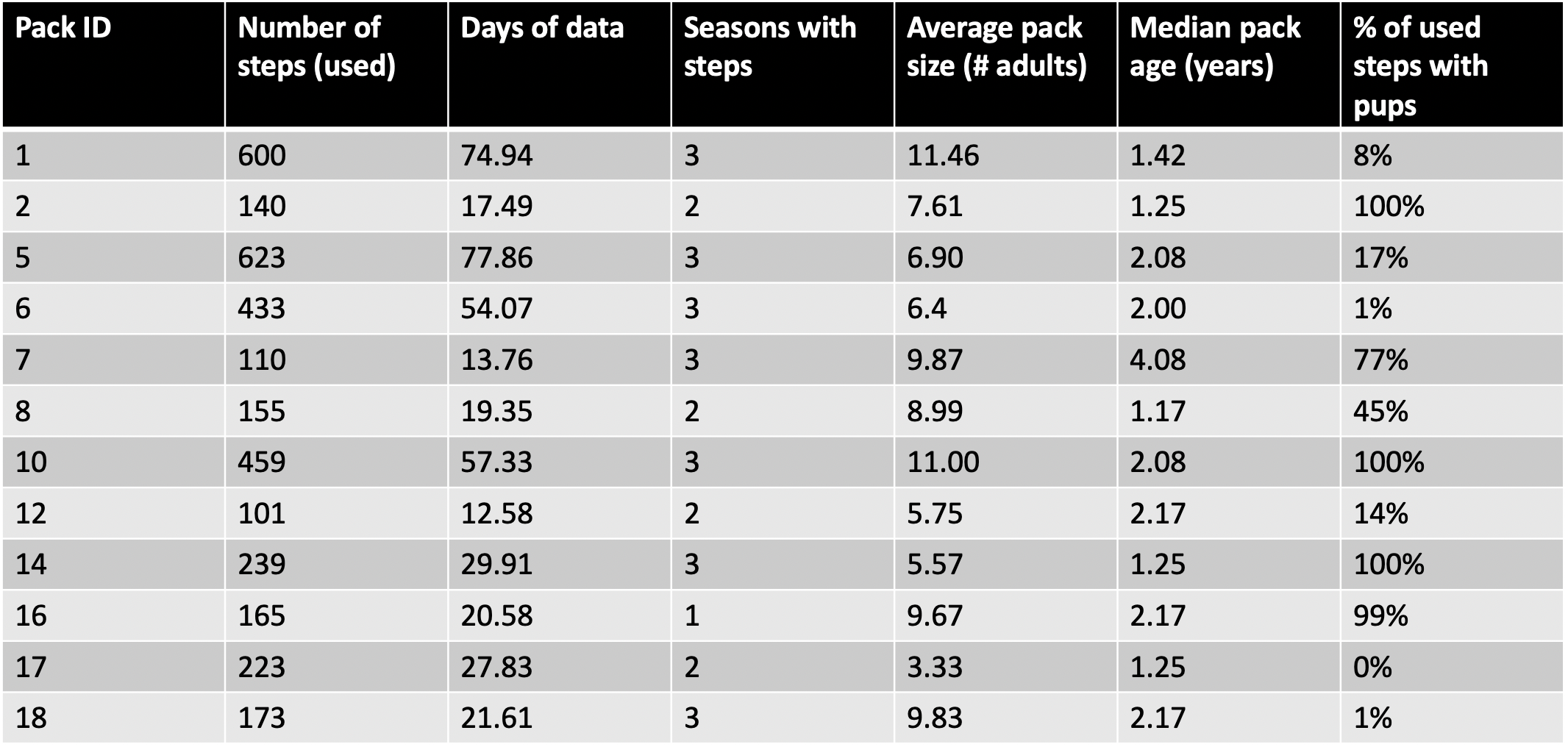
Table 6 illustrates the number of used steps per group, along with the number of days of data (number of used steps divided by 8, as steps occurred every 3 hours), the average pack size over all points, median pack age over all points, and percent of steps with pups. Note that “Days of data” does not mean cumulative days comprised of cumulative steps.
